# T54. TAILOR – TAPERED DISCONTINUATION VERSUS MAINTENANCE THERAPY OF ANTIPSYCHOTIC MEDICATION IN PATIENTS WITH NEWLY DIAGNOSED SCHIZOPHRENIA SPECTRUM DISORDERS IN REMISSION OF PSYCHOTIC SYMPTOMS

**DOI:** 10.1093/schbul/sby016.330

**Published:** 2018-04-01

**Authors:** Signe Dolmer, Mai Nielsen, Merete Birk, Ole Mors, Anne Emilie Stürup, Nikolai Albert, Heidi Dorthe Jensen, Lene Eplov, Carsten Hjortshøj, Bjørn Epdrup, Merete Nordentoft

**Affiliations:** 1 Aarhus University Hospital; 2 Copenhagen University Hospital, Mental Health Center Copenhagen

## Abstract

**Background:**

Schizophrenia spectrum disorders have major implications for the individuals, their families and society.

Antipsychotic medication is the cornerstone in the treatment of psychotic symptoms and is effective in the reduction of psychotic symptoms and of relapse after remission of psychotic symptoms. This is the reason for recommending maintenance treatment with antipsychotic medication in national and international guidelines for the treatment of schizophrenia, one year after remission of psychotic symptoms in first episode psychosis.

The aim of the study is to investigate the effect of tapered discontinuation versus maintenance therapy with antipsychotic medication in patients with newly diagnosed schizophrenia or persistent delusional disorder and with minimum three months remission of psychotic symptoms, and to find minimal effective dose of antipsychotic medication. Negative symptoms, cognitive impairments and the side effects of antipsychotic medication can cause a serious and long-term burden for patients and can reduce their quality of life. The TAILOR study will investigate these important aspects.

**Methods:**

The study is a randomized multicenter single blinded clinical trial. The aim is to include 250 patients from the outpatient early intervention program, OPUS, a 2 years manualized psychiatric treatment programme. At baseline patients must have 3 months remission of psychotic symptoms as documented by the SAPS (Schedule for Assessment of Positive Symptoms in Schizophrenia).

The patients will be randomized to either tapered discontinuation or dose reduction of antipsychotic medication or treatment as usual stratified according to substance abuse. The intervention will last for 1 year, and follow up interviews will be made after 1,2 and 5 years.

The patients will receive a user-developed mobile phone application to make daily registrations.

**Results:**

The study has been including patients since May 2017.

The first data is expected in 2019.

**Discussion:**

The TAILOR trial will contribute to knowledge about the effect of tapering/discontinuation of antipsychotic medication in early phases of schizophrenia spectrum disorders and hopefully the results may guide future clinical treatment regimens of antipsychotic medication.

The trial is a complex medical intervention, and it raises ethical, practical and organizational challenges.

When designing the TAILOR trial ethical questions were raised regarding blinding and the design of the intervention. In the trial only the researchers are blinded, neither clinicians nor patients, because they should be attentive of the high risk of relapse in the discontinuation group. The design gives the clinicians the possibility to adjust the dose of the antipsychotic medication to ensure sufficient treatment. Therefore, the trial only includes assessor blinding and the groups might end up being more similar than intended.

In general, it is of ethical consideration that the trial participants in the tapering/discontinuation group will be subjected to a higher risk of relapse. On the other hand, it seems unethical if research were not to discover the group of patients who can discontinue antipsychotic medication without relapsing.

Practical challenges will be sufficient recruitment or patient motivation and dropout.

